# Renal oncocytoma: experience of Clinical Urology A, Urology Department, CHU Ibn Sina, Rabat, Morocco and literature review

**Published:** 2012-07-24

**Authors:** Marwane Andaloussi Benatiya, Ghizlane Rais, Mounir Tahri, Ali Barki, Hachem El Sayegh, Ali Iken, Yassine Nouini, Azzouz Lachkar, Lounis Benslimane, Hassan Errihani, Mohammed Faik

**Affiliations:** 1 Clinical Urology Department A, CHU Ibn Sina, Rabat, Morocco; 2 Medical Oncology Department, National Institute of oncology, Rabat, Morocco

**Keywords:** Renal oncocytoma, tumor, diagnosis, treatment

## Abstract

Renal oncocytoma is a rare and benign renal tumor. Only few cases have been reported in Moroccan populations. In the present study, we report our experiences in the diagnosis, management and follow-up of this disease. We report on six cases of renal oncocytoma indentified between 1990 and 2008 in the urology department of “CHU Ibn Sina” in Rabat. These six cases are listed among 130 kidney tumors reported during the study period. We assess the clinical, radiological and therapeutic features of the patients and we review literature. Six cases of renal oncocytoma, representing 4.6% of all primitive kidney tumors treated in our institution during the study period. The mean age was 53 ±9.7 years (range 34 to 61 years). One patient was asymptomatic at presentation, five patients (83%) had flank pain and two (33%) had macroscopic hematuria. The tumor was right sided in 4 cases (66%) and left sided in 2 cases (33%). All patients underwent CT scan which showed, in three cases, a centrally located stellate area of low attenuation. The clinical suspicion of oncocytoma was made preoperatively in only 3 patients by imaging studies, but the suspicion of renal cell carcinoma persist and all patients were treated with radical nephrectomy. Definitive diagnosis was made in all cases postoperatively. All the tumors were well circumscribed but unencapsulated. The mean tumor size was 8,75±2,04 cm. Four patients were classified at stage pT2 and two at stage p T1. Most of the pathological features in our patients were typical of this entity. Predominant cell type was a typical oncocytoma with general low mitotic activity. No extension to peri-nephric fat tissue or lymphovascular invasion was observed. After a mean follow-up of 36 months (range 26-62 months), there was neither recurrence nor death from oncocytoma. Accordingly, the disease-specific survival was 100%. Renal oncocytoma has a benign clinical course with excellent long-term outcomes. In our series, it happened mostly in females and is more frequently symptomatic. Although radical nephrectomy is the usual treatment, a conservative approach should be considered whenever there are signs of clinical and radiological presumptions.

## Background

Oncocytoma is a rare renal tumor first described by Zippel in 1942 [1]. Renal Oncocytomas (RO) are usually solitary masses and represent between 3% and 10% of all renal tumors [2, 3]. Oncocytoma is considered to be a benign neoplasm in the majority of cases; this is the reason why there is only one documented case of liver metastasis in literature [4]. Clinically, oncocytoma may be asymptomatic, but symptomatic patients may present initial signs of haematuria, flank pain or palpable mass Oncocytomas usually appear to be unifocal, but multifocal and bilateral appearance and concomitant renal cell carcinoma (RCC) have been reported [2, 4].

The diagnosis of these benign lesions is generally suggested by computed tomography (CT) or magnetic resonance imaging (MRI). The appearance of a typical central stellate scar can occasionally be mimicked by necrosis in a renal cancer, this feature is not considered specific [5–7]. Moreover, fine needle aspiration and biopsy are often not diagnostic due to oncocytoma having similar histopathologic characteristics as various eosinophilic variants of renal cell carcinoma (RCC) [8]. Histologically, oncocytomas consist of round-to polygonal-shaped cells with an abundant finely granular cytoplasm [2, 9]. Careful attention to pathologic features and the adjunctive use of immunostains can aid in discriminating oncocytoma from other renal tumors characterized by granular, eosinophilic cytoplasm, especially chromophobe renal cell carcinoma [8]. Therefore, RO pose a special diagnostic and therapeutic challenge. Since no safe preoperative diagnostic differentiation between oncocytomas and RCC can be achieved, most patients undergo radical nephrectomy. However, nephron-sparing and laparoscopic surgical approaches (partial nephrectomy, enucleation or wegde resection) can be used to treat appropriately selected patients [4, 9]. The primary aim of this analysis is to outline the clinico-epidemiological, therapeutic profile and outcomes of patients with renal oncocytoma in our institution.

## Methods

We reviewed 130 primary nonurothelial epithelial renal neoplasms with primary resection at the Department of Urology A of the CHU IBN SINA (Rabat, Morocco) between the years 1990 and 2008 and classified 6 cases (4,6%) as renal oncocytomas. All patients underwent preoperative CT scan, in order to assign clinical stage according the TNM 2002 Staging System [10]. Pathological staging was assigned in accordance with the same staging system [10]. Data were collected from patient medical files and, clinicopathologic data including patient characteristics, clinical manifestations, surgical technique, pathologic findings and clinical outcome were analyzed. The detailed histopathological report was reviewed. Tumor size was evaluated by measuring the largest diameter of the tumor mass removed surgically. Every 6 months, patients underwent a periodical follow-up which included a physical examination, routine laboratory evaluation, ultrasounds and CT scan.

## Results

**Figure 1:**
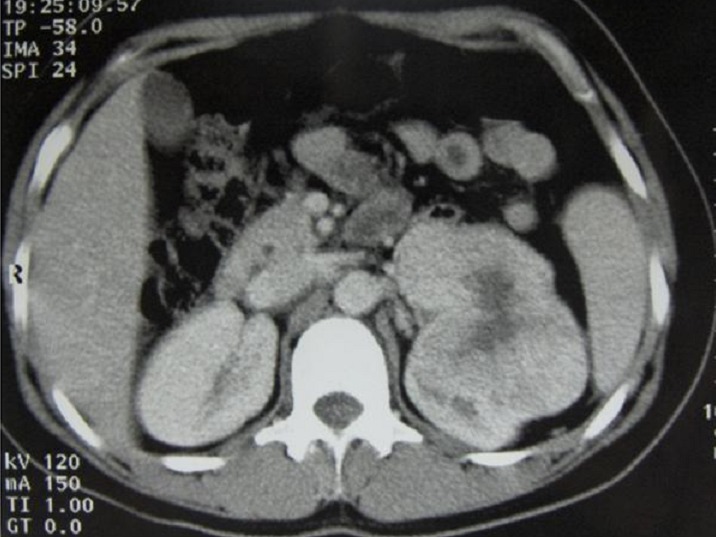
Computed tomography, shows a left renal tumor with central stellate scar

The study population was composed of a man and five women, and the mean age was 53 ±9.7 years (range 34 to 61 years). Only One patient was asymptomatic at presentation and was diagnosed incidentally as renal tumor by sonography. Five patients (83%) had flank pain and two (33%) had macroscopic hematuria. Physical examination was normal in all patients. The tumor was right sided in 4 cases (66%) and left sided in 2 cases (33%). No bilateral or multifocal disease was observed. All patients underwent CT scan which showed a centrally located stellate area of low attenuation in three of them (Figure 1). Preoperatively, the clinical impression of oncocytoma had been considered in only 3 (50%) patients following various imaging studies. However, other radiologic features suspecting RCC were noted and patients underwent radical nephrectomy. Definitive diagnosis was assessed in all cases postoperatively.

All the tumors were well circumscribed but unencapsulated. Tumor size ranged from 6,5 cm to 12 cm in greatest dimension, with a mean tumor size of 8.75±2.04 cm. Four patients were classified in stage pT2 and two in stage p T1. Most of the pathological features of the studied oncocytoma were typical of this entity. Surgical specimens were well-circumscribed, with no encapsulated masses, mahogany brown or pale yellow in color. Predominant cell type was a typical oncocytoma with general low mitotic activity. No extension to perinephric fat tissue or lymphovascular invasion was observed. After a mean follow-up of 36 months (range 26-62), there was neither recurrence nor death from oncocytoma. Accordingly, the disease-specific survival was 100%. The demographic and clinical features of the patients are summarized in Table 1.

**Table 1:** Demographic characteristics and clinical features of the patients

		No	%
Sex	Male	1	16.6
	Female	5	83.3
Laterality	Right	4	66.6
	Left	2	33.3
Mean age (years)		53±9.7 (34-61)	
Nephrectomy type	Radical	6	100
	Partial	0	0
Surgical approach	Open	6	100
	Laparoscopy	0	0
Presentation	Incidental	1	16.6
	Symptomatic	5	83.3
Pathological T stage	T1b	2	33.3
	T2	4	66.6
Mean pathological size (cm)		8.75 ±2.04 (6.5-12)	

## Discussion

Renal oncocytomas (RO) occur with an overall incidence of 3–10% among all renal tumors and are detected by chance mostly [2, 3]. Comparatively, our study shows that the incidence was 4.6% of the primary resectable renal masses in a 19-year period. RO occur more frequently in men than in women with male/female ratio of 2-3:1, and peak occurrence is between the ages of 40 and 60.have approximately the same mean age incidence as RCC [11]. The demographic characteristics of our population (Table 1) were slightly similar to those ones of previous reports [4, 12, 13]. Similarly, in our series, the mean age at diagnosis was 53 years; however there was a female predilection with a male-to-female ratio of 1:5.

Most of renal oncocytomas are asymptomatic at presentation and are discovered incidentally during evaluation for nonurological problems, whereas hematuria and pain occur in a minority of patients. The reported incidence of incidentally discovered masses varied from 50% [14] to 100% [15]. In our series, only one patient was asymptomatic, five patients had pain and two had hematuria (due probably to the large size of tumor at diagnosis).

RO usually appear as solitary lesions measuring 4-8 cm. The mean size of tumor was 8,75cm in our report, which is quietly larger than previously reported in the literature [13–16]. Multifocal and bilateral appearance have been observed in 4–6% and 4%, respectively [3, 12]. Accordingly, a multifocal and bilateral growth pattern is rare. No case of bilateral or multifocal oncocytoma was observed in our series.

No safe preoperative diagnostic differentiation between oncocytomas and RCC can be achieved because of the lack of pathognomonic radiographic signs. CT-scans show oncocytomas to be more homogenous than RCC. It usually reveals a solid homogeneous lesion with a centrally located stellate area of low attenuation; however, this is considered nonspecific and occurs in only 33% of oncocytomas [4]. On MRI, most oncocytomas demonstrate low signal intensity relative to the renal cortex on T1-weighted images [2]. Twenty-seven percent of oncocytomas also demonstrate a central stellate scar on MRI [17]. Angiography sometimes reveals the typical spoke-wheel formation; Bioptic procedures contain the risk of seeding tumor cells and cannot establish a safe distinction as well [18]. In our series, 3 patients presented with typical CT findings especially with a centrally located stellate area. However, these characteristics may suggest but cannot definitively diagnose oncocytoma [5, 19]

Recently, few articles have reported the use of positron emission tomography (PET) for the diagnosis of oncocytoma [20, 21]. Blake and al reported a case of RO displaying intense activity on 18F-fluorodeoxyglucose PET [20], but it is important to recognize that oncocytomas can yield false-positive results on 18F-FDG PET, and absolute radiologic differentiation of oncocytoma from RCC remains elusive. Finally, the novel imaging techniques were investigational, and further validated studies are required. We think that the diagnostic process may be complex and it could involve both imaging and bioptical assessment [2, 6, 7, 22]. To date, controversies still remain as far as exact radiological characterization of renal masses. Even if preoperative distinction between oncocytoma and RCC is considered as a critical step to avoid aggressive surgical procedures, to date any imaging technique can really distinguish between oncocytoma and malignant lesions [2]. In facts, it is widely recognized that the presence of renal clear cell carcinoma (RCC) with oncocytic features may occur, and there may be a frequent coexistence of RCC in the same or contralateral kidney of patients [23].Thus, we believe percutaneous core biopsy or fine needle aspirations play a very limited role in identification of renal neoplasm, but in certain circumstances, biopsy could definitively establish diagnosis [22].

Histologically, renal oncocytomas are typically well-circumscribed and often encapsulated [2, 9]. A central white-colored scar is occasionaly observed, especially in larger tumors[2, 4]. Microscopically, RO consist of a pure population of oncocytes, which are large well-differentiated neoplastic cells with intensely eosinophilic granular cytoplasm due to the large number of mitochondria [9, 24–25]. Like chromophobe carcinomas, oncocytomas appear to originate from collecting duct cells [25]. In most cases, oncocytomas and different histological subtypes of RCC can be differentiated on gross inspection and from H&E-stained microscopic slides. Sometimes the differentiation is difficult, especially that among the eosinophilic variant of chromophobe RCC, the granular variant of conventional RCC, and oncocytoma [26, 27].

With regard to Hales's colloidal iron stain, chromophobe RCC show strong diffuse positivity and oncocytoma show negative or weak focal staining. Recent data proposed that cytokeratin staining profiles may be useful for discriminating oncocytoma from its renal mimics: oncocytomas are typically CK7–, CK14+ and CK20+, while most chromophobe RCCs are positive for CK7; the various eosinophilic RCC are typically negative for CK14, and only 0–8% of RCCs are positive for CK20 [28, 29]. In our series, no immunohistochemichal staning was realized, because histological features were typical and in accordance with other similar reports.

Despite their benign behavior, oncocytomas should be monitored closely and treated if there is evidence of coexisting RCC, which occurs in 10% to 32% of reported patients [2], or if it had rapid growth with a risk of destroying the adjacent renal parenchyma [30].

Surgical treatment of RO is still unclear. Although radical surgery has been employed in the past as principal therapy, more precise preoperative and peri-operative diagnosis should allow more frequent use of conservative surgery, such as partial nephrectomy or tumor excision. Currently, nephron-sparing surgery has been recommended as the standard of care for renal oncocytoma. These include nephronsparing surgery, cryoablation, radiofrequency ablation, high intensity focused ultrasound, microwave thermotherapy and interstitial photon irradiation. Actually, cryo and radiofrequency ablation are the most studied. They have been performed laparoscopically and percutaneously[11]. Renal oncocytoma was not specifically considered as a leading diagnostic differential in our series, because oncocytomas are fairly rare in our context. Therefore, all patients underwent radical nephrectomy, but we think that conservative surgery, as possible, should be adopted in future cases.

## Conclusion

Renal oncocytoma has a benign clinical course with excellent long-term outcomes. In our series, it happened mostly in females and is more frequently symptomatic. As the overwhelming majority of cases behave in a benign fashion, conservative surgery is the mainstay of treatment, especially for patients with small tumors. However, as preoperative diagnosis based on imaging studies was uncertain, radical nephrectomy was warranted for all patients in our series.
